# Structural Characterization of a Novel *Chlamydia pneumoniae* Type III Secretion-Associated Protein, Cpn0803

**DOI:** 10.1371/journal.pone.0030220

**Published:** 2012-01-17

**Authors:** Chris B. Stone, Seiji Sugiman-Marangos, David C. Bulir, Rob C. Clayden, Tiffany L. Leighton, Jerry W. Slootstra, Murray S. Junop, James B. Mahony

**Affiliations:** 1 M. G. DeGroote Institute for Infectious Disease Research, Faculty of Health Sciences and the Department of Pathology and Molecular Medicine, McMaster University, Hamilton, Canada; 2 Father Sean O'Sullivan Research Centre, St. Joseph's Healthcare, Hamilton, Canada; 3 Department of Biochemistry and Biomedical Sciences, McMaster University, Hamilton, Canada; 4 Pepscan Presto, Zuidersluisweg, Lelystad, The Netherlands; University of Osnabrueck, Germany

## Abstract

Type III secretion (T3S) is an essential virulence factor used by Gram-negative pathogenic bacteria to deliver effector proteins into the host cell to establish and maintain an intracellular infection. *Chlamydia* is known to use T3S to facilitate invasion of host cells but many proteins in the system remain uncharacterized. The *C. trachomatis* protein CT584 has previously been implicated in T3S. Thus, we analyzed the CT584 ortholog in *C. pneumoniae* (Cpn0803) and found that it associates with known T3S proteins including the needle-filament protein (CdsF), the ATPase (CdsN), and the C-ring protein (CdsQ). Using membrane lipid strips, Cpn0803 interacted with phosphatidic acid and phosphatidylinositol, suggesting that Cpn0803 may associate with host cells. Crystallographic analysis revealed a unique structure of Cpn0803 with a hydrophobic pocket buried within the dimerization interface that may be important for binding small molecules. Also, the binding domains on Cpn0803 for CdsN, CdsQ, and CdsF were identified using Pepscan epitope mapping. Collectively, these data suggest that Cpn0803 plays a role in T3S.

## Introduction

*Chlamydia pneumoniae* is an obligate, intracellular Gram-negative bacterium associated with pneumonia and bronchitis. Members of the *Chlamydia* genus all share a unique, biphasic life cycle initiated by attachment of the metabolically quiescent elementary body (EB) to a host cell. The remaining intracellular portion of the life-cycle takes place within a plasma-membrane derived vacuole known as an inclusion. Once inside the inclusion, EBs transform into metabolically active reticulate bodies (RB) that becomes associated with the inclusion membrane. Interaction with the inclusion membrane allows RBs to communicate with the host cell via T3S, permitting *Chlamydia* to commandeer host cell pathways to acquire lipids, cholesterol, and other nutrients crucial for growth and replication [1]–[3]. RB replication results in expansion of the inclusion until an unknown stimulus signals non-infectious RBs to transform into infectious EBs which exit the host cell either by cell lysis or a packaged released mechanism termed extrusion, leaving the host cell intact [4].

T3S is a virulence mechanism used by several Gram-negative bacteria including *Yersinia*, *Salmonella*, *Pseudomonas* and *E. coli* to inject effector proteins from the bacterial cytosol into the host cell cytoplasm. The type III secretion system (T3SS) translocates effectors through the inner membrane, periplasmic space, and outer membrane in a single-step using a syringe-like apparatus known as an injectisome [5]–[7]. This apparatus is activated upon host cell-contact, possibly through interaction of the T3S injectisome with cholesterol and sphingolipid rich microdomains, termed lipid rafts, in the host cell membrane [8], [9]. Insertion of hydrophobic translocator proteins into host cell membranes is thought to be dependent on lipid-rafts since in the absence of cholesterol, translocators do not form pores and lyse artificial membranes [10]. The needle-filament protein YscF (*Yersiniae*) extends from the bacterial outer-membrane and houses the needle-tip complex containing the sensor protein and possibly the translocators [11]. The hierarchy of effector secretion at the inner membrane is thought to be controlled by T3S ATPases. The T3S ATPases (YscN orthologs) function at the inner membrane, interacting with T3S effector proteins to dissociate them from their cognate chaperones and allow effector passage through the injectisome [12]. Another protein that exists in both the soluble and insoluble fractions of the T3S system is the C-ring protein (YscQ orthologs), which may function as a platform to recognize effector/chaperone complexes, subsequently shuttling them to the inner membrane ATPase [13], [14]. The needle-tip complex, located at the tip of the T3S injectisome, is crucial for sensing host-cell contact and initiating secretion [15]. Needle-tip proteins first recognize cell contact and act as an extracellular chaperone facilitating translocator insertion into the host membrane [16], [17]. Crystallographic analysis of needle tip proteins revealed a dumbbell-like structure with two globular domains on either end of a “grip” formed by a conserved coiled-coil motif [18]. Based on comparisons of biophysical properties to other tip proteins, CT584 has been implicated as the needle tip protein of *C. trachomatis* [19]. At this time, nothing is known about the structure or function of Chlamydial needle tip proteins. Here, we present data demonstrating that Cpn0803 (CT584 ortholog) from *C. pneumoniae* interacts with several T3S components including the needle filament protein, the ATPase and the C-ring protein. We also mapped the regions of Cpn0803 responsible for mediating these protein interactions. Structure determination of Cpn0803 revealed a unique overall fold with no structural similarity on the DALI server. Taken together, this data suggests that Cpn0803 plays a role in *C. pneumoniae* type III secretion.

## Results

### Cpn0803 interacts with T3S proteins

**Figure 1:**
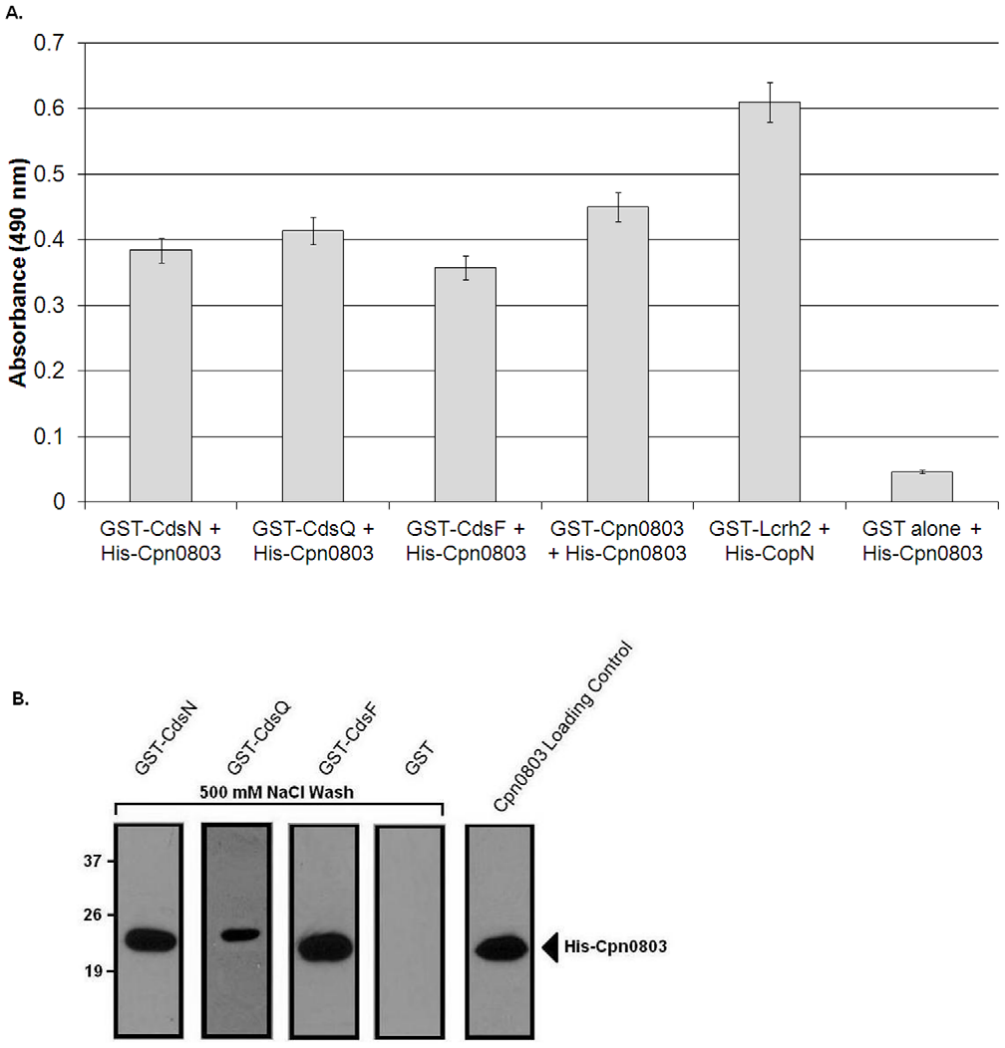
Cpn0803 interacts with type III secretion components *in vitro*. A. A glutathione plate assay was applied to screen for interactions of Cpn0803 between either CdsN, CdsQ or CdsF. His-Cpn0803 was applied to GST-CdsN, -CdsQ and -CdsF immobilized on glutathione plates, washed three times with PBS, and monitored using a colorimetric assay. Data is represented on the graph as the mean ± standard deviation for each interaction. A significant interaction was considered to be two standard deviations above the negative control (GST alone against Cpn0803). As a positive control, we screened GST-Cpn0803 against His-Cpn0803, as well as GST-Lcrh-2 against His-CopN. Cpn0803 interacted significantly with CdsN, CdsQ, CdsF, and Cpn0803. while it did not interact with GST alone. GST-Lcrh-2 and His-CopN also had a significant interaction. B. We applied GST pull-down assays to corroborate the interactions found with the glutathione plate assay. GST-CdsN, -CdsQ, –CdsF or GST alone immobilized on glutathione beads were incubated with an *E. coli* lysate over-expressing His-Cpn0803 and washed with 500 mM NaCl. GST-CdsN, -CdsQ, and CdsF co-purified with Cpn0803 under 500 mM NaCl conditions while GST alone did not.

**Figure 2:**
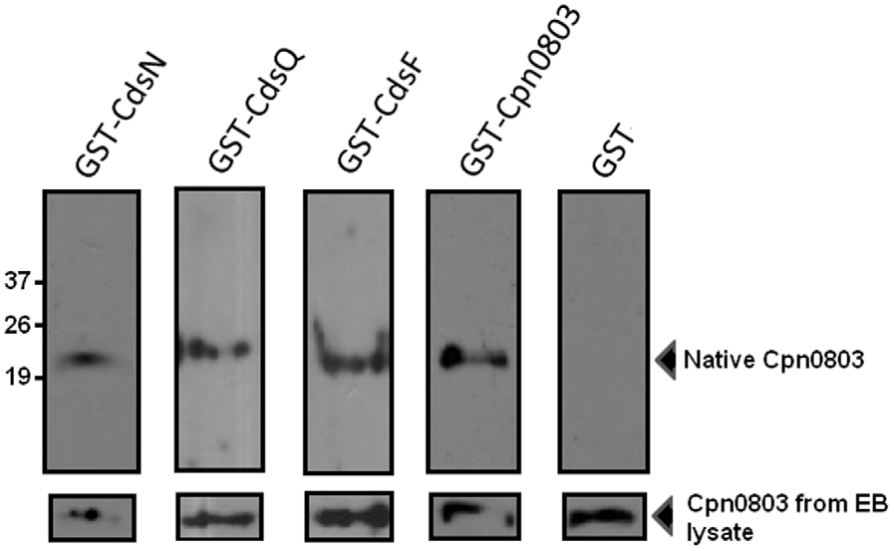
Cpn0803 interacts with type III secretion components *in vivo*. *C. pneumoniae* EB lysates were incubated with recombinant GST-CdsN, -CdsF, -CdsQ or -Cpn0803. Glutathione agarose beads were incubated with the lysates overnight, collected, and washed with 500 mM NaCl. The protein on the beads was analyzed by SDS PAGE and Western blot with anti-Cpn0803 antibody. Native Cpn0803 co-purified with GST-CdsN, -CdsF, and -CdsQ. As a positive control, Cpn0803 also co-purified with GST-Cpn0803, but not with GST alone.

To explore whether Cpn0803 interacts with other T3S proteins, we used ELISA and GST pull-down assays to evaluate possible protein interactions. First, GST-CdsN, GST-CdsQ and GST-CdsF were immobilized on glutathione plates, reacted against His-Cpn0803, and monitored using a colorimetric assay to evaluate possible protein interactions. Cpn0803 interacted with GST-CdsN, GST-CdsQ and GST-CdsF with absorbance values corresponding to 0.38±.008, 0.41±.006 and 0.36±.01 absorbance units, respectively, that were significantly higher than background levels (Figure 1A). As a positive control, we demonstrated that GST-Cpn0803 interacted with His-Cpn0803 with an absorbance of 0.45±.032. We also included a second positive control between GST-Lcrh-2 and His-CopN (absorbance of 0.61±.065), which have been shown to interact in previous studies [20]. Significant interactions were considered to be two standard deviations above the negative control (GST alone), which had an absorbance value of 0.046±.003. Next, CdsN, CdsF and CdsQ immobilized on glutathione beads were mixed with *E. coli* lysates containing His-Cpn0803. The beads were harvested by centrifugation and analyzed for His-Cpn0803 protein by anti-his Western blot. In each case, His-Cpn0803 co-purified with GST-CdsN, GST-CdsF or GST-CdsQ under high (500 mM) NaCl conditions, suggesting that the interaction is specific (Figure 1B). GST alone did not co-purify with Cpn0803 under any condition. To corroborate the *in vitro* GST pull-down assays, we used recombinant GST-CdsN, GST-CdsQ and GST-CdsF to pull-down native Cpn0803 from an EB lysate (Figure 2). We found that GST-CdsN, -CdsQ and -CdsF co-purified with native Cpn0803 from an EB lysate, suggesting that these proteins interact *in vivo*. As a positive control, GST-Cpn0803 co-purified with native Cpn0803 from an EB lysate while GST alone, as a negative control, did not.

### Structure of Cpn0803

**Figure 3:**
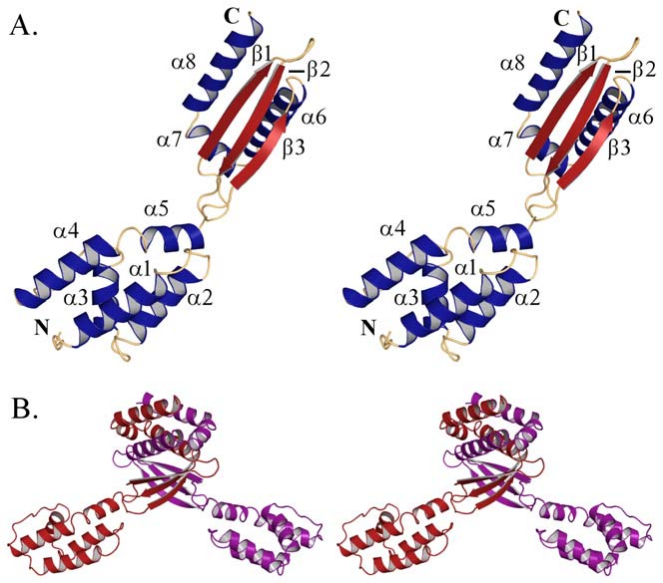
Stereo image of Cpn0803 monomer and dimer. The structure of full-length Cpn0803 was determined by SAD phasing of an anomalous data set collected from crystals of SeMet derivatized protein. The overall structure of Cpn0803 has a unique fold and no structural orthologs on the DALI server. A. Cartoon representation of the Cpn0803 monomer. Secondary structure elements are colored as follows; α-helices in blue and β-strands in red. B. Cartoon representation of the Cpn0803 dimer colored by chain.

The crystal structure of full-length Cpn0803 was determined by SAD phasing of an anomalous data set collected from SeMet derivatized protein (Figure 3). Cpn0803 crystallized in the hexagonal space group P6_3_, with two monomers in the asymmetric unit. The final model was refined to R and R_free_ values of 17.58 and 20.85%, respectively. Cpn0803 is 184 amino acids in length, with the final model spanning residues 8–182 in chain A and residues 8–183 in chain B. A complete list of X-ray diffraction data and model refinement statistics can be found in Table 1.

**Table 1:** Data Collection and Model Refinement Statistics.

		SeMet	Native
**Data collection**			
Space group		P6_3_	P6_3_
Cell parameters			
	a,b,c (Å)	85.28, 85.28, 97.83	86.39, 86.39, 99.59
	α, β, γ (°)	90, 90, 120	90, 90, 120
Molecules in ASU		2	2
Resolution (Å)^a^		50.0 – 2.30	50 – 2.00
Unique reflections		35, 361	28, 547
Redundancy^a^		4.4 (4.5)	9.7 (9.2)
Completeness (%)^a^		100.0(100.0)	100.0 (100.0)
I/σ(I)^a^		10.75 (2.15)	23.06 (4.10)
*R*_merge_ (%)^a^		12.1 (72.8)	9.1 (66.9)
Wilson B		-	26.19
**Model refinement**			
Resolution (Å)^a^			43.196 – 2.00
*R*_work_/*R*_free_ (%)			17.74/20.85
Reflections_observed_			27, 704
Reflections_Rfree_			1, 416
No. atoms			
	Protein		2, 953
	Ligand/ion		0
	Water		245
R.m.s.d. bond			
	Lengths (Å)		0.009
	Angles (°)		1.233
Average B Factor (Å^2^)			36.79
PDB Accession Code			3Q9D

a Statistics for the highest resolution shell are shown in parentheses.

The N-terminal 97 residues of the Cpn0803 monomer assembled into a 4-helix bundle in which the fourth helix is bent by a proline at position 87 (Figure 3). A short, five amino acid loop joins the N-terminal helical domain to a 3-stranded anti-parallel β-sheet. This β-sheet has one surface exposed to the solvent while the other is flanked by three α-helices. Helices α6 and α8 run anti-parallel to one another and are joined by a short, two turn helix (α7) sitting perpendicular to both which forms a ‘П’ shaped motif.

**Figure 4:**
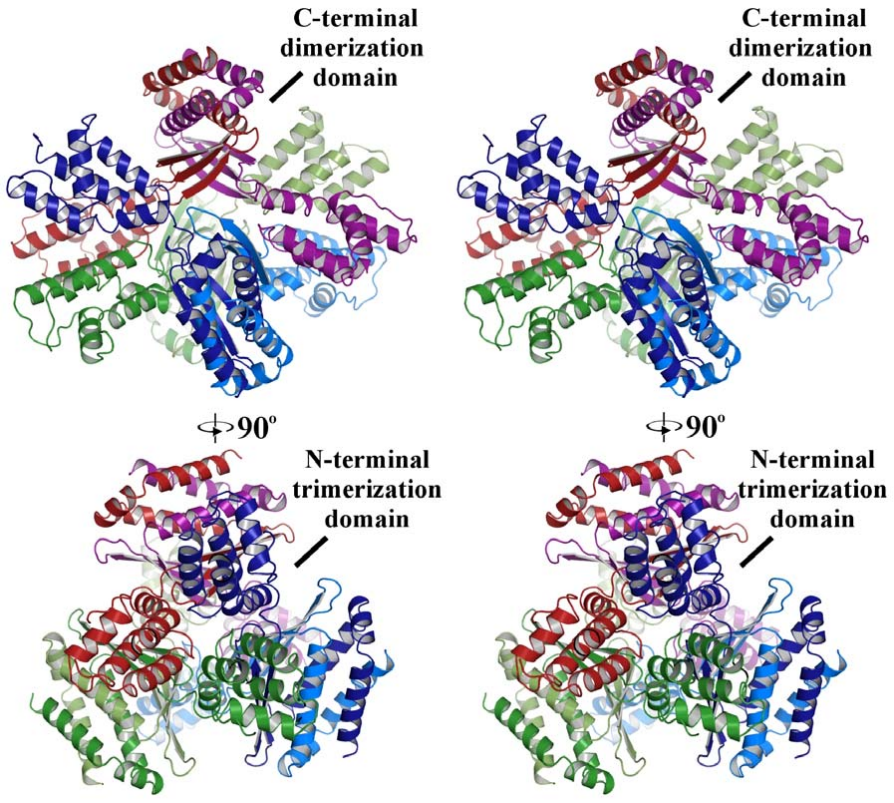
Stereo image of a predicted Cpn0803 hexamer colored by chain. We evaluated Cpn0803 for its ability to form multimers by analysis with the PISA server. Based on crystal contacts and buried surface area, the biologically active unit of Cpn0803 was predicted to be a hexamer formed by a trimer of dimers. The individual monomeric units are shown in different colours.

PISA is a software package able to predict the oligomeric structure of a protein based on crystal contacts [21]. PISA analysis suggested the biologically active unit of Cpn0803 to be a hexamer formed by a trimer of dimers (Figure 4). In the crystal structure, Cpn0803 forms a dimer mediated primarily by the C-terminal 3-helix motif. This interface involves 68 residues from each monomer and buries 6, 430 Å^2^, corresponding to approximately 35% of the total surface area of the complex. Helices α6 and α8 from either monomer associate to form a 4-helix bundle capped on either end by α7. This interaction is stabilized through many hydrophobic interactions, extensive hydrogen bonding and a number of salt bridges. In the N-terminal domain, a total of 21 residues from α5 and the loop region joining β2 and β3 form extensive interactions with two symmetry-mates in the crystal packing that result in the formation of a trimer, burying an additional 3, 867 Å^2^.

**Figure 5:**
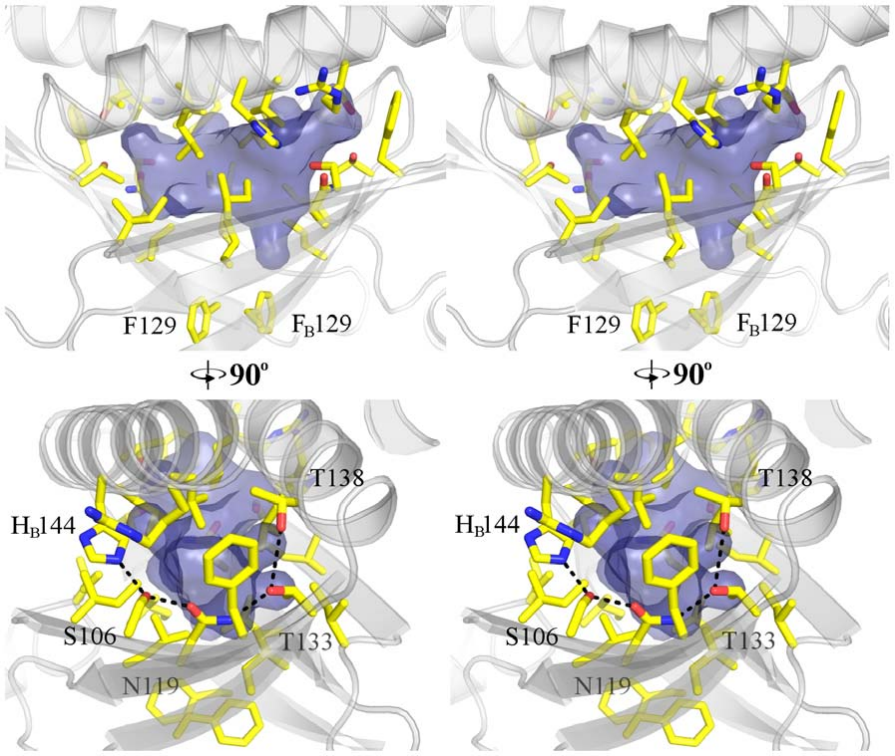
Stereo image of the hydrophobic pocket in Cpn0803. Dimerization of Cpn0803 results in the formation of a cone-shaped hydrophobic pocket between the two 3-stranded β-sheets, closed off at the ‘top’ by α-6 from both chains A and B, and stoppered at the ‘bottom’ by F129. The amino acid residues lining the interior of the pocket are represented in stick form.

Dimerization of Cpn0803 forms a cone-shaped hydrophobic pocket between the two 3-stranded β-sheets and is closed off at the ‘top’ by α6 from both chains A and B (Figure 5). This pocket is lined almost entirely with hydrophobic residues and is stoppered at the ‘bottom’ by F129 which forms a π-stacking interaction with its counterpart from the second monomer. Five polar residues lie on either side of the pocket, however, it appears that they are within close enough proximity to form a network and satisfy the hydrogen-bonding potential of their polar atoms as no ordered water molecules were observed within this hydrophobic cavity. Specifically the hydrogen-bond distances of T138–T133, T133–N119, N119–S106, and S106–H_B_144 are 3.4, 2.8, 2.5, and 2.7 Å respectively. All but T128–T133 are within ideal hydrogen bonding distance, and T128–T133 can still be considered a stabilizing electrostatic interaction if not a true hydrogen-bond. The pocket is approximately 15 Å in its longest dimension and has a volume of ∼211.3 Å^3^ as calculated by the CASTp server [22].

### Cpn0803 interacts with host cell lipids

**Figure 6:**
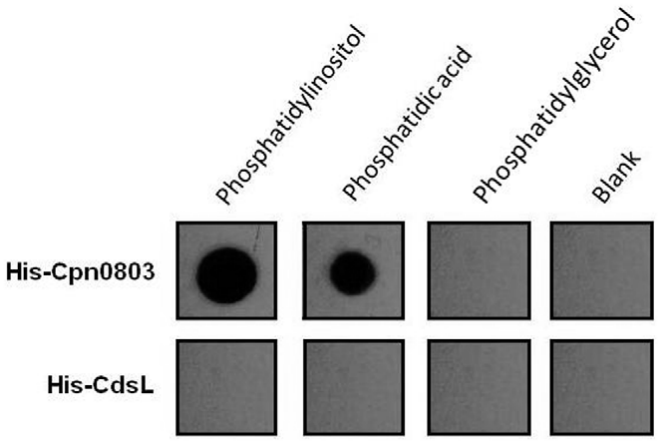
Cpn0803 interacts with phosphatidylinositol and phosphatidic acid. His-Cpn0803 was incubated with membrane lipid strips containing purified eukaryotic membrane components and visualized by anti-his antibody and ECL reagents. Cpn0803 interacted with phosphatidylinositol and phosphatidic acid, but none of the other molecules evaluated. His-CdsL, as a negative control, did not interact with any lipid components.

The presence of a hydrophobic pocket in Cpn0803 suggests that it may have small molecule binding partners. To explore whether Cpn0803 interacted with lipid components of the cell membrane, we incubated recombinant His-Cpn0803 with membrane lipid strips which contain small amounts of purified lipids immobilized on the surface of the strips. A positive interaction was detected by anti-His Western blot after incubation of His-Cpn0803 with the strips (Figure 6). These strips were used to analyze potential interactions with each of the following membrane components: triglycerides, diacylglycerol, phosphatidic acid, phosphatidylserine, phosphatidylethanolamine, phosphatidylcholine, phosphatidylglycerol, cardiolipin, phosphatidylinositol, PtdIns(4)P, PtdIns(4,5)P2, PtdIns(3,4,5)P3, cholesterol, sphingomyelin, and sulfatide. Cpn0803 interacted with two of the 15 lipid components evaluated, *viz*. phosphatidic acid (PA) and phosphatidylinositol (PI). His-CdsL (the T3S ATPase tethering protein and negative regulator) was used as a negative control and did not react with any of the lipid components.

### Cpn0803 exists as a hexamer in solution

**Figure 7:**
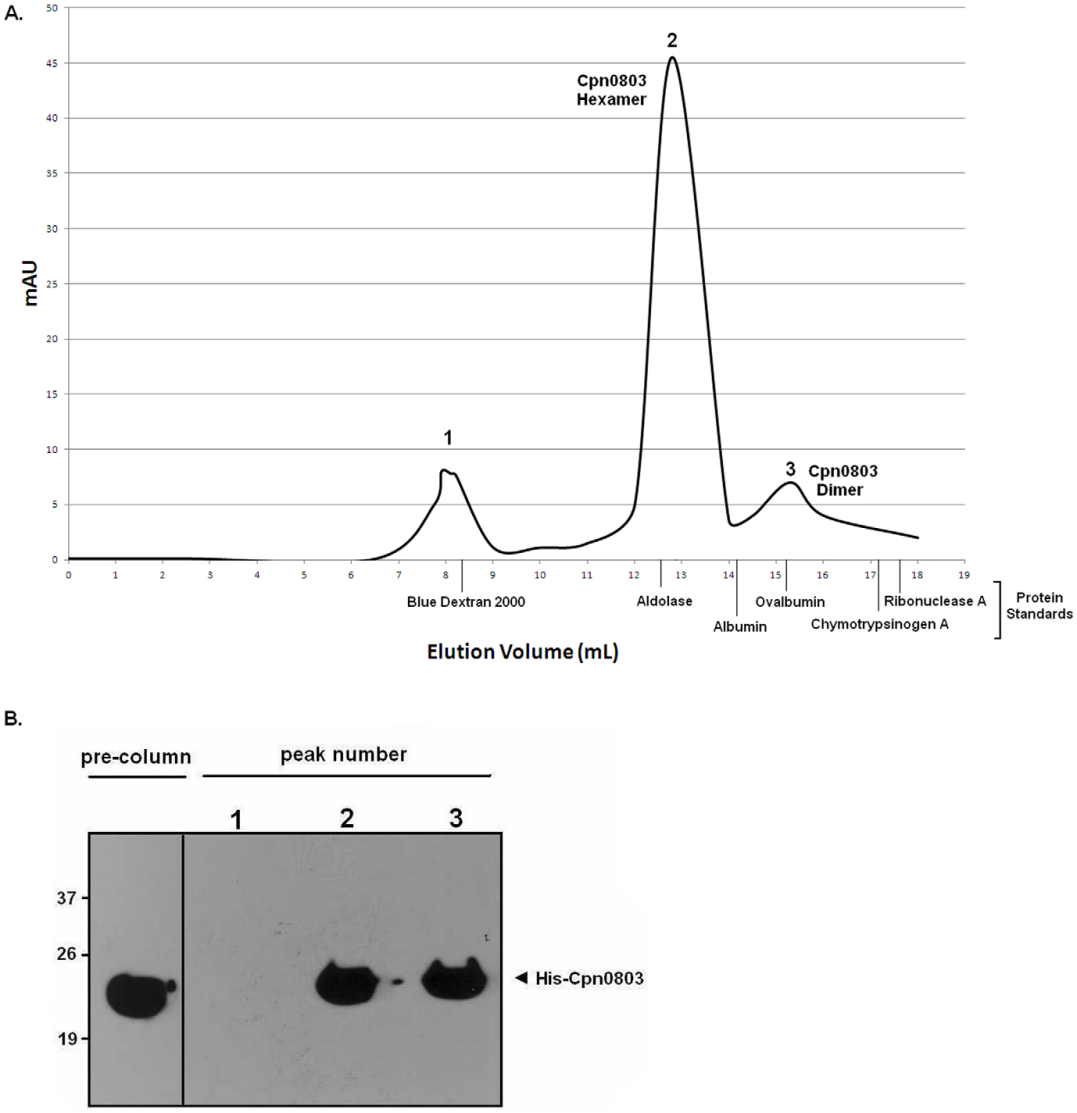
Cpn0803 exists in a hexameric and dimeric state in solution. A. Gel filtration chromatography was used to analyze purified His-Cpn0803. We found two dominant peaks, one corresponding to a hexamer and one corresponding to a dimer. The elution volume of the protein standards are shown on the x-axis. B. Peak fractions in the elution volumes were collected and analyzed by anti-His Western blot. Cpn0803 was present in the peak fractions.

To explore whether Cpn0803 formed higher-ordered structures, we analyzed purified recombinant Cpn0803 using size-exclusion chromatography (Figure 7). Prior to performing gel chromatography, the column was standardized using the LMW and HMW gel filtration standard kit. The elution volume of the protein standards are shown on the x-axis. Cpn0803 eluted as a predominant peak corresponding to a hexamer, with a smaller peak corresponding to a dimer (Figure 7A). Both peaks were shown to contain Cpn0803 as determined by anti-His Western blot (Figure 7B).

### Mapping of protein binding domains on Cpn0803

**Figure 8:**
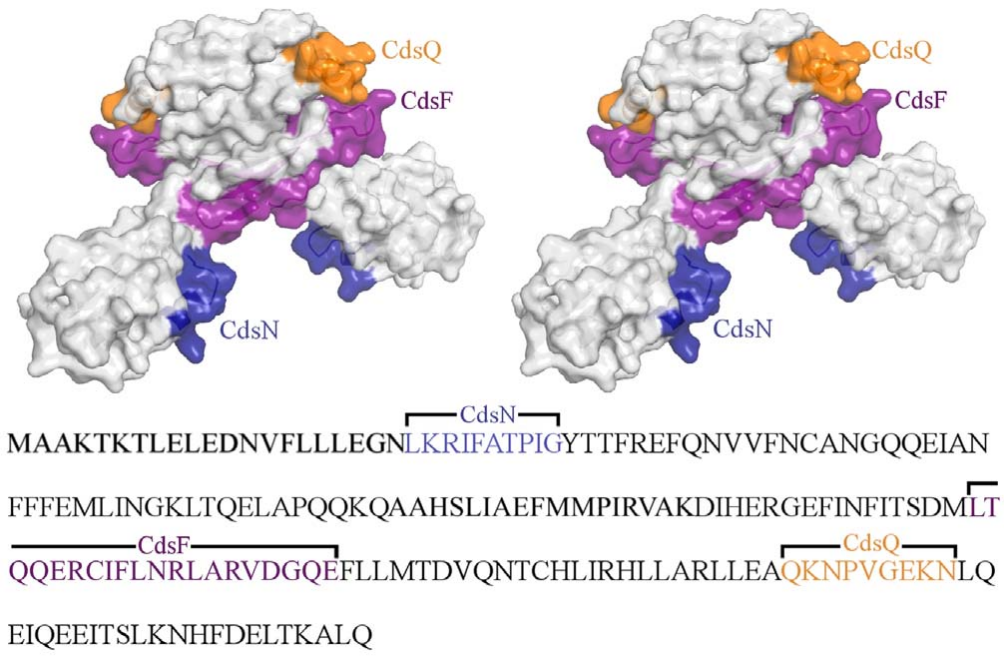
Pepscan mapping of the Cpn0803 binding regions shown in stereo. A. Pepscan epitope mapping against a Cpn0803 peptide library was performed to determine the residues of Cpn0803 responsible for mediating its interactions with CdsN, CdsF and CdsQ. Recombinant CdsN, CdsF and CdsQ was reacted against the Cpn0803 peptide library and monitored for the corresponding interacting regions. The corresponding surfaces are color-coded as follows: CdsN in blue (residues 22–26), CdsF in purple (residues 109–128), and CdsQ in orange (residues 153–161).

**Figure 9:**
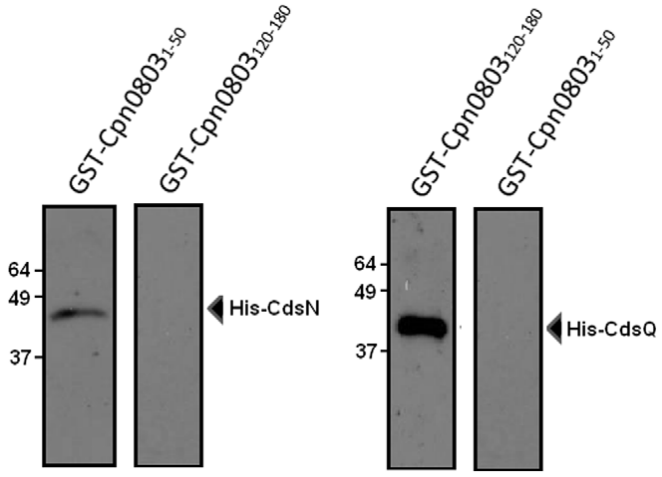
Corroboration of the Cpn0803 binding regions identified by Pepscan using GST pull-downs. To corroborate the Pepscan results, we expressed 50 amino acid fragments of Cpn0803 encompassing the CdsN and CdsQ binding domains and performed GST pull-down assays against recombinant CdsN or CdsQ. GST-Cpn0803_120–180_, containing the CdsQ binding domain, co-purified with His-CdsQ under high salt conditions. GST-Cpn0803_1–50_, which does not contain the CdsQ binding domains, did not co-purify with CdsQ. GST-Cpn0803_120–180_, which does not contain the CdsN binding domain, did not co-purify with CdsN.

Having shown that Cpn0803 interacts with CdsN, CdsQ, and CdsF, we next mapped the Cpn0803 binding surfaces for these proteins. We used Pepscan epitope mapping for this purpose, as has been described previously [23], [24]. This method can identify protein binding domains in Cpn0803 by screening binding partners against a library of 13 amino acid Cpn0803 peptides. Thus, an overlapping peptide library of Cpn0803 was synthesized and incubated with GST-CdsN, His-CdsQ, or His-CdsF (Figure 8). Pepscan epitope mapping revealed distinct binding domains on Cpn0803 for CdsN, CdsQ, and CdsF. Specifically, CdsQ binding was localized to a surface corresponding to residues QKNPVGEKN (amino acids 153–161) in the C-terminal region (Figure 8). The CdsN binding surface corresponded to residues LKRIFATPIGYTTFR (amino acids 22–36) (Figure 8) at the N-terminus, and the CdsF binding surface corresponded to residues LTQQERIFLNRARVDGQE (amino acids 109–129). Random peptides sequences from Cpn0803, ranging from 15–30 amino acids in length, were used as negative controls and did not interact with CdsN, CdsQ, or CdsF in the assay. To corroborate the Pepscan epitope mapping, we cloned and expressed fragments of Cpn0803 containing the CdsQ and CdsN interacting regions fused to GST and performed GST pull-down assays against His-CdsQ and His-CdsN (Figure 9). GST-Cpn0803_1–50_, containing the CdsN binding domain, co-purified with His-CdsN under high salt conditions. GST-Cpn0803_120–180_, containing the CdsQ binding domain, co-purified with His-CdsQ under high salt conditions. GST-Cpn0803_1–50_, which does not contain the CdsQ binding domains, did not co-purify with CdsQ under any conditions. GST-Cpn0803_120–180_, which does not contain the CdsN binding domain, did not co-purify with CdsN under any conditions.

## Discussion

Although *C. pneumoniae* contains a functional T3SS, the identification and function of several key proteins of the apparatus have not been elucidated. In this report, we characterized a novel T3S-associated protein Cpn0803 in *C. pneumoniae*, demonstrating interactions with several key proteins of the T3SS. Using ELISA and co-purification assays, we show that Cpn0803 interacts with the ATPase CdsN, the needle filament protein CdsF, and the C-ring protein CdsQ, and mapped their respective binding surfaces using Pepscan mapping. We also show that Cpn0803 interacts with eukaryotic cell membrane components PA and PI, suggesting that Cpn0803 may associate with the host cell. The crystal structure of Cpn0803 revealed a relatively large, isolated, hydrophobic pocket within the dimer interface which may play a role in binding small molecules such as PA and PI. These observations suggest that Cpn0803 plays a role in T3S and may associate with host-cell components.

Chlamydial T3S genes are encoded on ten operons scattered throughout the genome [25]. The fact that Cpn0803 is not encoded on one of the ten operons does not preclude the possibility that Cpn0803 is a T3S effector or chaperone protein. For example, TARP (a known T3S effector) is encoded separately from the ten operons, not clustered with other T3S components. Thus, we evaluated binding of Cpn0803 to T3S membrane and peripheral-membrane components. T3S ATPases (YscN orthologs) promote effector protein unfolding and secretion by dissociating their cognate chaperones [12]. We have previously shown that CdsN is the ATPase of the *C. pneumoniae* T3SS and binds several effectors and chaperones, and the proposed C-ring protein CdsQ [26]. Thus, the interaction between Cpn0803 and CdsN is expected if Cpn0803 is secreted through the injectisome. Spaeth *et al*. have recently presented data that supports a role for CdsQ in *C. trachomatis* as a platform that organizes effector/chaperone complexes at the basal body of the T3S apparatus [14]. Thus, the interaction of Cpn0803 with CdsQ is also consistent with a possible role as a T3S effector or chaperone protein. The interaction of Cpn0803 with the needle filament protein, CdsF, suggests that Cpn0803 may function at the tip of the injectisome and also supports its role in T3S. Taken together, these observations suggest that Cpn0803 may function as an effector or chaperone protein in *Chlamydia* T3S.

Cpn0803 crystallized in the hexagonal space group P6_3_, with two molecules in the asymmetric unit. A DALI search with the structure of Cpn0803 yielded no significant hits to any proteins in the current structural databases. Since CT584 (Cpn0803 ortholog) was predicted to be the needle-tip protein in *C. trachomatis* based on biophysical comparisons, we evaluated whether the structure of Cpn0803 had any similarity to needle-tip orthologs. Cpn0803 shares very little amino acid orthology, secondary or tertiary structure similarity with LcrV or IpaD. The only shared characteristic between these three proteins is a 4-helix bundle at the N-terminus. However, the central coiled-coil motif, which is conserved in all needle-tip families, is absent from Cpn0803 [15]. Based on both PISA analysis of the crystal structure and size-exclusion chromatography data of the recombinant protein, Cpn0803 appears to exist in a dimer/hexamer equilibrium in solution. Based on the crystal structure, the Cpn0803 hexamer is globular and approximately 9.5 nm in diameter in its longest dimension. However, the subunits are tightly packed and do not form a pore. To function as an LcrV ortholog, the oligomeric state of the needle-tip must permit passage of effector proteins through the T3S apparatus [27]. Thus, the oligomeric state and crystal structure of Cpn0803 does not support its role as the needle-tip protein. However, it is possible that alternative oligomeric forms exist which contain a pore, possibly after translocation through the needle-complex.

We have shown that Cpn0803 interacts with both PA and PI. The volume of the hydrophobic pocket within the dimerization interface of the crystal structure of Cpn0803 is approximately 211.3 Å^3^, making this hydrophobic pocket large enough to bind a small molecule. PA and PI are approximately 415 and 196 Å^3^, respectively, indicating that PI, but not PA, could bind in the pocket. Conformational changes in the structure of Cpn0803 induced by oligomerization could lead to changes in the size of the hydrophobic pocket and facilitate binding of other small molecules. It is possible that Cpn0803 undergoes conformational changes upon binding PA or PI, and that this change is required for Chlamydial infection of host cells and effector translocation. *Chlamydia* is known to recruit proteins involved in phosphatidylinositol-4-phosphate (PI4) metabolism to regulate the PI composition of the inclusion membrane, which is necessary for optimal chlamydial development [28]. It is possible that Cpn0803 also plays a role in regulating PI levels in the inclusion. Also, phosphatidylinositol-3,4,5-P_3_ is synthesized at sites of chlamydial entry upon host cell infection and may be important for actin recruitment and activation of specific guanine nucleotide exchange factors (GEFs) [29]. Cpn0803 may play a role in regulating these GEFs within the host cell upon infection through its association with PI. Thus, the Cpn0803 interaction with PI supports a possible role as a T3S effector protein.

We employed Pepscan epitope mapping to determine the Cpn0803 binding surfaces involved in the interaction with CdsN, CdsQ, and CdsF [23], [24]. We found that Cpn0803 possesses specific surfaces mediating these interactions. The binding surface for CdsN corresponded to residues 22–36 on the N-terminal domain of Cpn0803. Based on the crystal structure, this surface would be buried within the hexameric state of Cpn0803. Therefore, cytoplasmic Cpn0803 likely interacts with CdsN as a dimer. Partial unfolding of the dimer would further permit secretion through the needle. CdsQ binding was mapped to a surface on Cpn0803 corresponds to residues 153–161 located at the C-terminus. The separation between the N-terminal CdsN and the C-terminal CdsQ binding surfaces may be important in the “hand-off” or transfer of Cpn0803 from the cargo transporter protein CdsQ to the ATPase CdsN. The CdsF binding surface on Cpn0803 corresponds to residues 109–129. This CdsF binding surface is partially inaccessible in the hexameric Cpn0803 state, but exposed in the dimer, suggesting that the Cpn0803 exists as a dimer upon interaction with CdsF. The observation that the interactions between Cpn0803 and CdsN/CdsF only occur with dimeric Cpn0803 suggests that the hexameric form may represent a stable cytoplasmic Cpn0803 structure, prior to inner membrane recruitment and subsequent secretion. The oligomerization may also be required for effector function in the host cell.

In the absence of a genetically tractable system for *Chlamydia*, and the inability to knockout Cpn0803, the definitive role of Cpn0803 cannot be determined. The epitope mapping of binding surfaces for key T3S proteins, such as the needle-filament protein CdsF and the ATPase CdsN, supports the role of Cpn0803 in type III secretion and possibly as an effector protein. However, the crystal structure of Cpn0803 has very little similarity to LcrV or IpaD orthologs and does not support its role as the needle-tip protein. Whether Cpn0803 represents a novel class of needle-tip proteins, or plays an alternative role in T3SS such as that of an effector protein, requires further investigation.

## Methods

### Expression Plasmids

*C. pneumoniae* CWL029 (VR1310:ATCC) (GenBank accession # AE001363) was used to isolate genomic DNA for cloning and protein expression. Full length *cdsN*, *cdsQ*, *cdsF*, *copN*, *lcrh-2*, and *cpn0803* were amplified from CWL029 using AttB-containing primers (Gateway; Invitrogen). Amplified products were cloned into pDONR_201_ (Gateway; Invitrogen) to generate pENT vectors which were then used in LR reactions (Gateway; Invitrogen) to produce pEX vectors containing genes of interest. Either pEX_17_ (N terminal His-tag) or pEX_15_ (N terminal GST-tag) vectors were used for protein expression. All constructs were confirmed by DNA sequencing.

### Purification of Recombinant Proteins

*E. coli* pellets containing over-expressed His- or GST-tagged proteins were thawed on ice and sonicated using a Fischer Scientific Sonic Dismembrator Model 100, followed by centrifugation at 20,000× g for 40 minutes to remove insoluble material. Supernatants containing His-tagged protein for use in GST pull-down assays were stored at 4°C. GST tagged protein for use in GST pull-down assays were bound to 300 µL of glutathione beads overnight at 4°C, then blocked overnight in Tris Buffered Saline with 0.1% Tween-20 and 4% BSA and stored at 4°C until use. Purity was assessed using SDS-PAGE and Coomassie blue staining. For His-tagged proteins, columns were washed with increasing imidazole concentrations and proteins were eluted with 300 mM imidazole then dialyzed extensively against activity buffer.

### Glutathione plate assays

The glutathione-plate assay was performed using 96-well Pierce Glutathione Coated Plates. Briefly, 200 µL of a lysate over-expressing a GST fusion protein was added to each well and incubated at 4°C for 2 hours. Next, the lysate was removed and the plate was washed with 200 µL of PBS five times. The plate was blocked using 4% BSA in TBST for 2 hours at 4°C, and then washed five times with 200 µL of PBS. Bacterial lysate (300 µL) over-expressing a poly-histidine fused protein was added to each well and incubated at 4°C for 2 hours. To disrupt non-specific interactions, the plate was washed eight times with 500 mM NaCl, 0.1% Triton X-100, 50 mM TRIS-HCl pH 7.5. After the wash, the wells were blocked with a 4% BSA solution in TBST for 45 minutes at room temperature. Mouse-anti-6×His monoclonal antibody (1∶1000 dilution in PBST) was incubated for 45 minutes at room temperature. Bound antibody was detected using a goat-anti-mouse horseradish peroxidase conjugate (1∶1000 dilution in PBST) and incubated at room temperature for 45 minutes, followed by 100 µL of 5 mg/mL OPD (o-Phenylenediamine) (Pierce) added to each well and incubated at room temperature for 30 minutes before reading the absorbance at 450 nm using a Bio-Tek ELx800 plate reader. An interaction was considered positive if it was two standard deviations above the negative control (GST alone).

### GST pull-downs

To examine the interaction of Cpn0803 with CdsN, CdsF, and CdsQ, GST pull-down assays were performed as described previously by Johnson et al, 2008, with the following modifications [30]. Briefly, glutathione-agarose beads (30 µL) bound to 50 ng of GST-tagged protein was used in the assay. The beads were incubated overnight at 4°C with the *E. coli* lysate expressing the His-tagged proteins. Beads were collected by centrifugation and washed with 0.1% Triton X-100 and increasing concentrations of NaCl to eliminate spurious protein interactions. The presence of GST- and His-tagged proteins was confirmed by both Coomassie blue and Western blot. GST alone bound to glutathione beads was used as a negative control for the pull-down.

### Production and affinity purification of rabbit polyclonal antibody to Cpn0803

Hyper-immune rabbit antisera was raised against Cpn0803. Briefly, His-Cpn0803 was expressed in *E. coli* BL21 and purified on Ni nitrilotriacetic acid (NTA) agarose (Qiagen) according to established procedures, and 500 µg was delivered to Cocalico Biologicals, Inc. (Reamstown, PA), as a Coomassie-stained sodium dodecyl sulfate-polyacrylamide gel electrophoresis (SDS-PAGE) gel slice. Three rounds of immunizations with the adjuvant Titermax were employed. Immunoglobulin G (IgG) molecules were precipitated from the hyper-immune antisera with 50% saturated ammonium sulfate solution, resuspended in cold phosphate-buffered saline (PBS), and dialyzed into PBS. Activated CH-Sepharose beads (Sigma) were coupled with 30 mg of His-Cpn0803and used to affinity purify the rabbit anti-Cpn0803 IgG. Briefly, antibody was incubated with the His-Cpn0803-conjugated beads overnight at 4°C with rocking and washed with ice-cold PBS until the *A*_280_ was less than 0.02. Glycine (100 mM, pH 3.0) was used to elute the rabbit anti-Cpn0803 IgG molecules in 1 ml fractions into Eppendorf tubes containing 10 µl of 1.5 M Tris, pH 8.8. Fractions were analyzed by probing Western blots containing chlamydial EB lysate and an *E. coli* lysate containing overexpressed His-Cpn0803.

### Co-purification assays

Co-purification assays were performed essentially as described previously to investigate the interaction of Cpn0803 from EBs with GST-tagged T3S proteins [30]. Briefly, 72 hr postinfection, EBs were purified on a discontinuous gradient and lysed in PBS containing 1% Triton X-100 for 1 hr. EB lysates were pre-cleared by microcentrifugation at 16, 000× *g* for 30 min and then incubated with GST-tagged proteins in PBS for 6 h. Gluathione agarose beads (20 µl) were then added to each tube overnight. Beads were collected by brief centrifugation, washed four times with binding buffer containing 20 mM imidazole, and boiled in 2× SDS-PAGE loading buffer. Co-purified Cpn0803 was detected by Western blotting and ECL using hyper-immune guinea pig anti-Cpn0803 antiserum.

### Crystal structure of Cpn0803

All Cpn0803 crystals were grown at 18°C using the hanging-drop vapour diffusion method. Equal volumes of Cpn0803 (3.0 mg/mL) in storage buffer (0.12 M potassium chloride, 0.2 M Tris pH 7.0) were mixed with crystallization solution (0.2 M potassium nitrate, 20% w/v PEG 3350) and dehydrated against 500 uL of 1.5 M ammonium sulfate. Cpn0803-SeMet crystals were grown in the same manner by streak seeding with native crystals and gradually increasing the concentration of ammonium sulfate in the well solution to 2.0 M.

Native and SAD (SeMet) data sets were collected at 1.1 and 0.979 Å wavelengths on beamlines ×29 and ×25 respectively of the National Synchrotron Light Source at Brookhaven National Laboratory. Data sets were processed and scaled with HKL2000 to 2.0 and 2.3 Å, respectively [31]. Of the 12 SeMet sites expected based on primary sequence, 11 were located by AutoSol in the anomalous data set. The Phenix software package was used to calculate initial phase information to generate an experimental map [32]. A preliminary model was built which was then used for Molecular Replacement into the higher resolution, native data set of Cpn0803 using AutoMR. Model building and structure refinement of Cpn0803 was carried out through multiple iterations of Phenix-Refine and manual manipulation in COOT until the values of R and R_free_ converged and geometry statistics reached acceptable ranges [33], [34]. Surface area calculations were performed with the PDBe PISA server and volume calculations were performed using Chimera and the CASTp server [21], [22]. All structural illustrations were generated with PyMol [35]. Crystal structure similarity predictions were obtained using the DALI server [36].

### Size-exclusion chromatography

Purified Cpn0803 (250 µl) in gel filtration buffer (20 mM Tris pH 7, 100 mM KCl, 5 mg/mL protein concentration) was injected into a Superdex S200 10/300 GL gel filtration column (Amersham Biosciences, Piscataway, New Jersey) at 0.1 mL/min. The column was standardized using the LMW and HMW gel filtration standard kit (GE Life Sciences). Elution fractions (1 mL) were collected at a flow rate of 1 mL/min. Peak fractions were examined by anti-His Western blot.

### Membrane lipid strip assay

Membrane lipid strips were purchased from Echelon and protein binding assays were performed according to the manufacturer's protocol. Briefly, lipid strips were blocked overnight in blocking buffer (PBS, 0.1% Tween, 5% BSA) at 4°C. Next, His-Cpn0803 or His-CdsL (negative control) were added to the lipid strips at a concentration of 0.5 µg/mL and incubated at room temperature for 1 h. The protein solution was discarded and the lipid strips were washed 5 times with PBS-T for 5 min. The lipid strips were then probed for bound His-tagged protein using anti-His antibody (1∶10000 dilution) and visualized with ECL reagent.
